# A comprehensive image dataset of Bangladeshi mango variety

**DOI:** 10.1016/j.dib.2025.111560

**Published:** 2025-04-15

**Authors:** Rup Kumar Bharati, Md. Masudul Islam, Md Ripon Sheikh, Galib Muhammad Shahriar Himel

**Affiliations:** a Department of Computer Science and Engineering, Jahangirnagar University, Dhaka, Bangladesh; b Department of Computer Science and Engineering, Bangladesh University of Business and Technology (BUBT), Dhaka, Bangladesh; c Department of physics, Jahangirnagar University, Dhaka, Bangladesh; d School of Computer Sciences, Universiti Sains Malaysia, 11800 USM Penang, Malaysia

**Keywords:** Mango variety, Deep learning, Image processing, Machine learning, Fruit classification, Mango classification, Agriculture, Image dataset

## Abstract

This data article presents a primary dataset collected from various locations in Bangladesh, featuring 10 different mango varieties that are mostly consumed locally. This mango dataset includes the following types: Amrapali, Bari-4, Bari-7, Fazlee, Harivanga, Kanchon Langra, Katimon, Langra, Mollika, and Nilambori. A DSLR camera was used to take high-resolution pictures of every mango variety; as a consequence, 2012 photographs were obtained, although the distribution of images among types is not uniform. This dataset, which provides a thorough representation of 10 distinct mango types, each with a distinct flavour, has a great deal of potential for impact and application. It offers a range of uses in the food production and agriculture sectors and offers insightful information for further study and development.

Specifications Table

**Table:** 

Subject	Computer Sciences
Specific subject area	*Computer Vision, Pattern Recognition, machine learning, deep learning*
Type of data	*JPEG Raw Image*
Data collection	*This dataset provides a collection of high-resolution images representing 10 widely consumed mango varieties from Bangladesh. Carefully selected from nearby markets and rural fruit farms, these mangoes guarantee a varied and representative collection. Serving as a visual compendium, the dataset facilitates precise classification by offering a comprehensive overview of these mango types. It includes 10 distinct classes: Amrapali, Bari-4, Bari-7, Fazlee, Harivanga, Kanchon Langra, Katimon, Langra, Mollika, and Nilambori, with a total of 2012 raw images. Each variety was systematically photographed between May 1 and August 1, 2024. This dataset is noteworthy for its novelty, as it represents a completely new resource that has not been utilized in any previous research.*
Data source location	***Location****: Dhaka* ***Country****: Bangladesh*
Data accessibility	Repository name: Mendeley Data Data identification number: 10.17632/w5jg84txj8.1 Direct URL to data: https://data.mendeley.com/datasets/w5jg84txj8/1
Related research article	

## 1 Value of the Data

• Researchers studying agriculture can use the dataset to examine the phenotypic characteristics of various mango cultivars. This can help distinguish and categories different varieties of mangos according to their outward appearance, including their size, shape, and texture. Improved farming practices may result from studies on how environmental factors affect mango appearance, which might be supported by the detailed photographs.
• The dataset is useful for machine learning method creation and testing, especially in the domains of image processing and computer vision. The data can be used by researchers to train models for automatic mango detection, categorization, and quality evaluation. The dataset is ideal for creating reliable algorithms that can be used in practical situations, such automated sorting and grading systems in the agriculture sector, because of its high-resolution photos and range of angles.
• Applications with a consumer focus, like smartphone apps for classifying mango types or rating their quality in stores, can be developed using this dataset. Developers are able to create tools that assist customers in making well-informed decisions about buying mangoes by utilizing the comprehensive pictures to highlight quality, ripeness, and variety factors.
• This dataset is a unique and comprehensive collection that can be utilized to investigate geographic variations in mango varieties across different locations within Bangladesh, or in comparison studies with other fruit datasets.
• This dataset offers a unique opportunity to advance machine learning applications in agriculture. Its potential spans from developing automated mango sorting systems to empowering consumers with mobile applications for mango variety identification. By enabling more precise classification and quality assessment, this dataset contributes to improving agricultural practices, fostering innovation in the food industry, and enhancing consumer decision-making.

## 2 Background

One of the most extensively grown and economically significant tropical fruits worldwide is the mango (Mangifera indica L.), which is mostly produced in South Asia, which produces a large amount of the world's mango crop [1]. Bangladesh is a major producer [2], along with India, and its mango varietals are prized for their distinct tastes, scents, and textures. Bangladesh boasts a wide range of mango cultivars, including well-known types like Amrapali, Langra, and Fazlee, all of which have unique qualities that make them suitable for different types of cooking and consumer tastes. In Bangladesh, the mango season usually lasts from May to August, when a wide variety of these types appear in the marketplaces. This is a critical time for export as well as domestic consumption, since the fruit is important to the nation's agricultural economy. To maintain and increase the market value of these mangoes, both locally and globally, research into their classification, quality evaluation, and post-harvest handling is crucial [3]. These efforts are supported by the availability of comprehensive datasets on mango types, which help with a better knowledge and optimisation of growing techniques.

## 3 Data Description

**Fig. 1:**
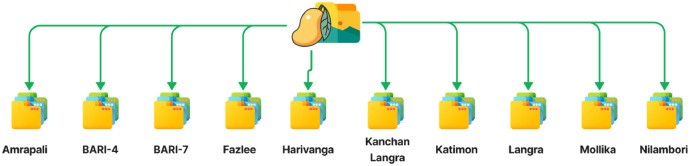
Dataset structure.

We present a dataset in this article that is arranged into a main folder that has ten individual sub-folders, each of which belong to a different kind of mango. 2012 high-quality JPEG files with a resolution of 3024×4032 pixels are contained in the root folder. The dataset has a significant file size of 3.04 GB because of its high resolution. The 'Mango_Dataset_2012.zip' ZIP file containing the dataset can be accessed via the Mendeley repository [4]. The ten sub-folders in this ZIP file are identified as follows: Amrapali, Bari-4, Bari-7, Fazlee, Harivanga, Kanchon Langra, Katimon, Langra, Mollika, and Nilambori. An illustration of the dataset's structure may be found in Fig 1. This dataset holds significant promise as a cutting-edge resource for developing machine vision algorithms aimed at classifying different mango varieties within the agricultural sector. Table 1 describes the physical and internal characteristics of each mango variety with example images.

**Table 1:** Mango variety description with mango images.

Mango Variety Name	Description
Amrapali	**Size and Shape:** Amrapali mangoes are medium-sized, typically weighing between 250–300 g They have an oval shape with a slightly pointed bottom and a rounded top. **Skin:** The skin is smooth and shiny, starting green when raw and turning yellowish-orange as it ripens. **Flesh:** The pulp is a vibrant yellowish-orange, soft, and fiberless, offering a rich, juicy taste with a sweet aroma. **Stone:** The fruit contains a small, flat stone in the center. **Color:** When ripe, the Amrapali mango’s skin transitions to a yellowish-orange hue, while the flesh remains a deep orange-red. **Texture:** The flesh is smooth and fiberless, making it easy to eat and enjoy. **Taste:** Known for its sweetness, the Amrapali mango has a rich, juicy flavor that is highly aromatic.
Bari-4	**Size and Shape:** Bari-4 mangoes are relatively large, typically weighing around 600 g They have a nearly round shape. **Skin:** The skin is smooth and yellow-green when ripe. **Flesh:** The flesh is deep yellow, fiberless, and very sweet, with a Brix value of 24.5 %, indicating high sugar content. **Stone:** The fruit contains a small, flat stone, with the flesh making up about 80 % of the fruit. **Color:** When ripe, the Bari-4 mango’s skin turns yellow-green, while the flesh becomes a bright orange. **Texture:** The flesh is soft, juicy, and fiberless, making it easy to eat and enjoy. **Taste:** Known for its delectable sweetness, the Bari-4 mango offers a perfect balance of sweetness and tanginess
Bari-7	**Size and Shape:** Bari-7 mangoes are medium-sized, typically weighing around 290 g They have a round shape, making them easy to handle and consume. **Skin:** The skin is smooth and has a very attractive yellow color with a reddish tinge when ripe. **Flesh**: The flesh is juicy, sweet, and fiberless, with a Total Soluble Solids (TSS) content of 18.3 %, indicating a high level of sweetness. **Stone:** The fruit contains a very small seed, maximizing the edible portion. **Color:** When ripe, the Bari-7 mango’s skin turns a vibrant yellow with a slight reddish blush, while the flesh remains a bright yellow. **Texture:** The flesh is smooth, juicy, and fiberless, making it highly enjoyable to eat**. Taste:** Known for its pleasant flavor, the Bari-7 mango offers a delightful balance of sweetness and juiciness.
Fazlee	**Size and Shape**: Fazlee mangoes are among the largest mango varieties, typically weighing between 500 g to 2 kg. They have an oval or oblong shape with a distinct pointed end. **Skin**: The skin is smooth and firm, starting green when unripe and turning pale yellow or light green as it ripens. **Flesh**: The flesh is firm, fiberless, and has a rich, sweet taste with a slight tanginess. It has a sugar content of 14–16 %, making it quite sweet. **Stone**: The fruit contains a relatively small seed compared to its size, maximizing the edible portion. **Color:** When ripe, the Fazlee mango’s skin transitions to a pale yellow or light green hue, while the flesh remains a vibrant yellow. **Texture**: The flesh is smooth and fiberless, providing a pleasant eating experience. **Taste**: Known for its unique sweetness with a hint of tanginess, the Fazlee mango also has a distinct aroma that enhances its appeal.
Harivanga	**Size and Shape:** Harivanga mangoes are typically round with a slightly elongated appearance. They usually weigh between 200 and 500 g, but some can reach up to 700 g **Skin**: The skin is smooth and dark green when unripe, turning slightly reddish as it ripens. **Flesh**: The flesh is juicy, sweet, and fiberless, offering a delightful eating experience. It is known for its rich, aromatic flavor. **Stone**: The fruit contains a small, flat stone, maximizing the edible portion. **Color**: When ripe, the Harivanga mango’s skin transitions from dark green to a slightly reddish hue, while the flesh remains a vibrant yellow. **Texture**: The flesh is smooth, juicy, and fiberless, making it highly enjoyable to eat. **Taste**: Known for its exceptional sweetness and rich aroma, the Harivanga mango is a favorite among mango lovers.
Kanchan Langra	**Size and Shape:** Kanchan Langra mangoes are medium-sized, typically weighing between 200 and 400 g They have an oblong or oval shape. **Skin:** The skin is green when unripe and turns yellowish-green as it ripens. It retains a greenish tinge even when fully ripe. **Flesh**: The flesh is soft, juicy, and fiberless, offering a rich, buttery taste with a pleasant aroma. **Stone**: The fruit contains a small, thin seed, maximizing the edible portion. **Color**: When ripe, the Kanchan Langra mango’s skin transitions to a yellowish-green hue, while the flesh remains a vibrant yellow. **Texture**: The flesh is smooth, juicy, and fiberless, making it highly enjoyable to eat. **Taste**: Known for its sweet and tangy flavor with a hint of honey, the Kanchan Langra mango is a favorite among mango enthusiasts
Katimon	**Size and Shape:** Katimon mangoes are medium-sized and oval-shaped, typically weighing between 200 and 300 g **Skin**: The skin is smooth and showcases vibrant yellow and orange hues when ripe. **Flesh**: The flesh is deep yellow-orange upon ripening. It is firm, creamy, and fiberless, offering a sweet flavor with a hint of tartness. **Stone**: The fruit contains a thin seed, maximizing the edible portion. **Color**: When ripe, the Katimon mango’s skin transitions to a bright yellow-orange, while the flesh becomes a deep yellow-orange. **Texture**: The flesh is firm and creamy, making it highly enjoyable to eat. **Taste**: Known for its sweet flavor with a slight tartness, the Katimon mango is a favorite among mango enthusiasts.
Langra	**Size and Shape:** Langra mangoes are medium to large in size, typically weighing between 200 and 400 g They have an oblong or oval shape with a slightly hooked tip. **Skin**: The skin is green when unripe and turns yellowish-green as it ripens. It retains a greenish tinge even when fully ripe. **Flesh**: The flesh is soft, juicy, and fiberless, offering a rich, buttery taste with a pleasant aroma. It has a sweet and tangy flavor with a hint of honey. **Stone**: The fruit contains a small, thin seed, maximizing the edible portion. **Color**: When ripe, the Langra mango’s skin transitions to a yellowish-green hue, while the flesh remains a vibrant yellow. **Texture**: The flesh is smooth, juicy, and fiberless, making it highly enjoyable to eat. **Taste**: Known for its sweet and tangy flavor with a hint of honey, the Langra mango is a favorite among mango enthusiasts.
Mollika	**Size and Shape:** Mallika mangoes are medium-sized, typically weighing between 250 and 500 *g* They have an oblong shape with a slight curve at the stem end. **Skin**: The skin is smooth and thin, transitioning from greenish-yellow to bright yellow-orange as it ripens. **Flesh**: The flesh is deep orange, firm, juicy, and fiberless. It offers a sweet and slightly tangy flavor with hints of citrus and melon. **Stone**: The fruit contains a small, narrow seed, maximizing the edible portion. **Color**: When ripe, the Mallika mango’s skin turns a bright yellow-orange, while the flesh remains a vibrant deep orange. **Texture**: The flesh is smooth, firm, and juicy, making it highly enjoyable to eat. **Taste**: Known for its sweet and aromatic flavor with a hint of tartness, the Mallika mango is a favorite among mango enthusiasts.
Nilambori	**Size and Shape:** Nilambori mangoes are medium to large in size, typically weighing between 300 and 500 g They have an oval shape with a slightly pointed end. **Skin**: The skin is smooth and thin, starting green when unripe and turning a bright yellow as it ripens. **Flesh**: The flesh is deep yellow, juicy, and fiberless, offering a sweet and slightly tangy flavor. **Stone**: The fruit contains a small, flat seed, maximizing the edible portion. **Color**: When ripe, the Nilambori mango’s skin transitions to a bright yellow hue, while the flesh remains a vibrant yellow. **Texture**: The flesh is smooth, juicy, and fiberless, making it highly enjoyable to eat. **Taste**: Known for its sweet and slightly tangy flavor, the Nilambori mango is a favorite among mango enthusiasts.

## 4 Experimental Design, Materials and Methods

**Fig. 2:**
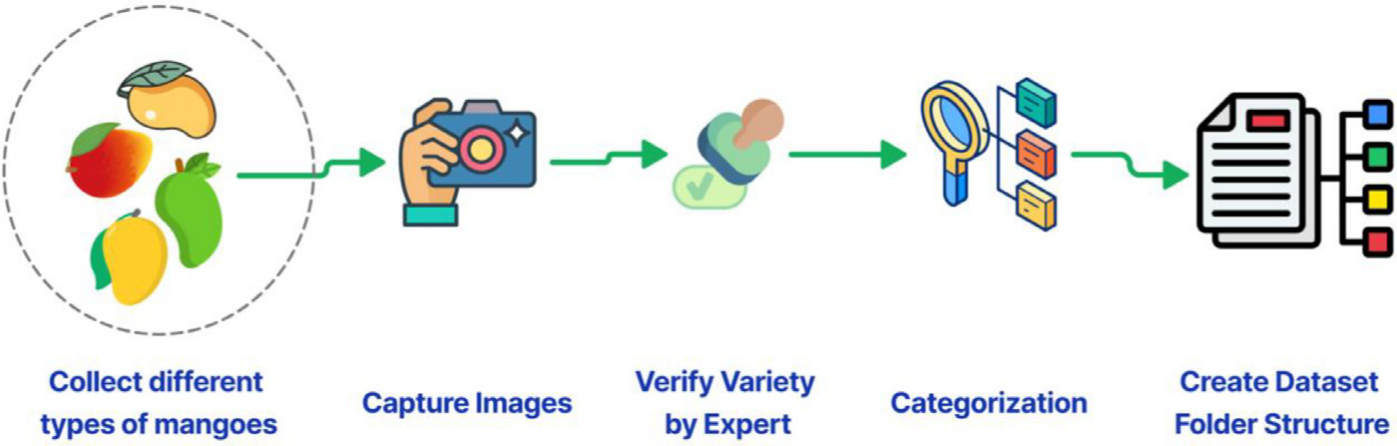
Mango image data collecting process.

Fig 2 illustrates the thorough approach that was followed during the picture acquisition phase for every variety of mango. Using a uniform distribution strategy, individual mangos were selected from each type's population to ensure a diverse dataset, giving each mango an equal chance of being chosen. In order to preserve data diversity, this random selection procedure was essential. Many difficulties were experienced when gathering the data. When mangos were purchased from naturally occurring trees and neighborhood markets, they frequently displayed different degrees of rot, partial worm or bat eating, or other damage. Mangos with defects were removed from the dataset as a result of these conditions, which presented serious challenges to the identification process. From a cluster of mangos, each mango selected for photography was done so at random. Once the photos were taken, they were moved from the memory of the camera to an external hard drive and arranged into folders that corresponded to the different varieties of mango. Local names were selected to label the data because they are more widely recognized than official ones, and agricultural specialists were contacted to guarantee accuracy. Only when the photographs from the previous variety of mango were cleared from the camera's memory did the process of taking images for the next type of mango begin. This methodical process was followed until photos of every category of mango—from raw to ripe—were acquired, guaranteeing a complete dataset. Our dataset's statistical overview is given in Table 2. To capture these images, we have used the Sony DSLR-A230Y which is a compact and lightweight digital SLR camera featuring a 10.2-megapixel APS-C CCD sensor, providing detailed and sharp images. It comes with a BIONZ image processor for fast performance and a user-friendly interface with an intuitive Quick AF Live View. The camera includes a dual lens kit (18–55 mm and 55–200 mm) for versatile shooting, and it supports SteadyShot INSIDE for in-body image stabilization.

**Table 2:** Details of mango varieties dataset.

Variety name	Number of original images	Variety name	Number of original images
Amrapali	252	Kanchon Langra	210
Bari-4	235	Katimon	163
Bari-7	176	Langra	202
Fazlee	yy	Mollika	221
Harivanga	202	Nilambori	195

## Limitations

There are >10 categories of mangoes available in Bangladeshi local market which were not included in this dataset limiting the number of categories. Another major limitation of the mango dataset is its uneven distribution across the 10 mango varieties, which could introduce bias in analysis and model training. Additionally, the dataset primarily includes mangoes sourced from local markets and naturally growing trees, which may not cover all possible variations present within the same category in both commercial and experimental settings. The presence of deformities in some mangoes was a challenge, leading to their exclusion and potentially limiting the dataset’s representativeness. Furthermore, the dataset only captures mangoes at specific ripeness stages, which may not fully represent the range of maturity levels seen in real-world scenarios.

## Ethics Statement

This article does not involve research with human or animal subjects by any of the authors. The datasets used in this article are publicly accessible, and proper citation guidelines should be followed when using these datasets.

## CRediT authorship contribution statement

**Rup Kumar Bharati:** Resources, Data curation. **Md. Masudul Islam:** Project administration, Conceptualization, Methodology, Formal analysis, Resources, Writing – original draft, Visualization. **Md Ripon Sheikh:** Resources, Data curation. **Galib Muhammad Shahriar Himel:** Project administration, Supervision, Conceptualization, Methodology, Formal analysis, Resources, Writing – original draft, Writing – review & editing.

## Data Availability

Mendeley DataMangifera2012: An Image Dataset of Various Bangladeshi Mangoes (Original data)
